# Comparison of superior and inferior vena cava diameter variation measured with transthoracic echocardiography to predict fluid responsiveness in mechanically ventilated patients after abdominal surgery

**DOI:** 10.1186/s12871-022-01692-8

**Published:** 2022-05-17

**Authors:** Qian Ma, Jingjing Ji, Xueduo Shi, Ziyun Lu, Lu Xu, Jing Hao, Wei Zhu, Bingbing Li

**Affiliations:** Department of Anesthesiology, Nanjing Drum Tower Hospital, The Affiliated Hospital of Nanjing University Medical School, 321 Zhongshan Road, Nanjing, 210008 Jiangsu China

**Keywords:** Superior vena cava collapsibility index, Inferior vena cava distensibility index, Superior vena cava cardiac variation, Fluid responsiveness, Transthoracic ultrasonography

## Abstract

**Background:**

The volume status of patients after major abdominal surgery constantly varies owing to postoperative diverse issues comprising fluid loss or capillary leakage secondary to systemic inflammatory reaction syndrome, *et.al*, the precise fluid responsiveness assessment is crucial for those patients. The purpose of this study is to validate the transthoracic ultrasonographic measurement of superior and inferior vena cava variation in predicting fluid responsiveness of mechanically ventilated patients after surgery.

**Methods:**

A total of 70 patients undergoing the scheduled major abdominal surgeries in the anesthesia ICU ward were included. The superior vena cava (SVC) collapsibility index (SVCCI), the inferior vena cava distensibility index (dIVC), SVC variation over the cardiac cycle (SVCV), and cardiac output (CO) were measured by transthoracic ultrasonography were recorded before and after fluid challenge test of 5 ml/kg crystalloid within 15 min. The responders were defined as a 15% or more increment in CO.

**Results:**

Thirty patients (42.9%) responded to fluid challenge, while the remnant forty patients (57.1%) did not. The areas under the ROC curve (AUC) of SVCCI, dIVC and SVCV were 0.885 (95% CI, 0.786–0.949; *P* < 0.0001) and 0.727 (95% CI, 0.608–0.827; *P* < 0.001) and 0.751 (95% CI, 0.633–0.847; *P* < 0.0001), respectively. AUC_dIVC_ and AUC_SVCV_ were significantly lower when compared with AUC_SVCCI_ (*P* < 0.05). The optimal cutoff values were 19% for SVCCI, 14% for dIVC, and 15% for SVCV. The gray zone for SVCCI was 20%-25% and included 15.7% of patients, while 7%-27% for dIVC including 62.9% of patients and 9%-21% for SVCV including 50% of patients.

**Conclusion:**

Superior vena cava-related parameters measured by transthoracic ultrasound are reliable indices to predict fluid responsiveness. The accuracy of SVCCI in mechanically ventilated patients after abdominal surgery is better than that of dIVC and SVCV.

**Trial registration:**

ChiCTR-INR-17013093. The initial registration date was 24/10/2017.

## Background

Rational goal-directed fluid management is one of the crucial components of anaesthesiologists' daily tasks. The latest multicentral retrospective study demonstrated that inappropriate fluid administration is correlated with increase in postoperative complications [1]. Thus, excessive fluid infusion may increase the risk of pulmonary and peripheral tissue edema retarding the recovery of respiratory and intestinal function, while the conservative fluid therapy may induce an unstable hemodynamic profile, multiorgan hypoperfusion, and prolonged hospital stay [2]. Furthermore, the volume status of the critically ill patients admitted to the anesthesia intensive care unit (AICU) after surgery is inevitably affected by diverse postoperative factors, including the over-zealous supplement of iv infusion, to some extent of urine loss, postoperative bleeding, abdominal drainage, and occult sweat evaporation [3]. Therefore, precise diagnosis and individualized fluid resuscitation tailored to every postoperative patient who is either over-or underhydration remain challenging.

Traditional static hemodynamic parameters, such as central venous pressure (CVP) and pulmonary capillary wedge pressure, have shown little value in guiding volume expansion [4]. In addition, although widely used and accepted as robust indicators to predict preload responsiveness in mechanically ventilated patients, the dynamic indices of stroke volume variation (SVV) [5] or pulse pressure variation (PPV) [6], require a costly sophisticated device or invasive catheterization [7]. With the increasing accessibility of ultrasound devices in perioperative settings, ultrasonography has been recommended for volume assessment due to its advantage in noninvasiveness, repeatability, and short learning curve [8, 9].

Among these echocardiographic variables, superior vena cava (SVC) collapsibility index (SVCCI), and SVC variation over the cardiac cycle (SVCV) have shown promising results in mechanically ventilated patients, but SVC measurements required transesophageal echocardiography (TEE) technique, which currently limited its routine clinical application [9–11]. With the introduction of SVC acquisition through transthoracic echocardiography (TTE) approach, it is, therefore, possible to determine volume responsiveness by measuring SVC variation using a noninvasive approach [12]. The objective of the present study was to evaluate and compare the predictive value of SVCCI and SVCV acquisition from transthoracic ultrasound with inferior vena cava distensibility index (dIVC) in fluid responsiveness for mechanically ventilated patients following abdominal surgery.

## Methods 

### Ethics approval and consent to participate

The Institutional Ethics Committee of Nanjing Drum Tower Hospital approved this trial (No.2017–122-02) that was registered in the Chinese Clinical Trial Registry (registration number: ChiCTR-INR-17013093, registration date: 24/10/2017). All patients provided written informed consent. All methods were carried out in accordance with the relevant guidelines and regulations of Nanjing Drum Tower Hospital.

### Patient populations

From September to December 2020, the prospective observational study was conducted in the AICU of a tertiary teaching hospital. Mechanically ventilated patients who need further treatment in the AICU after major abdominal surgery, aged 25 to 75 years, conforming to the American Society of Anaesthesiologists physical status II to III were recruited in this study. Patients with spontaneous respiratory effort, cardiac arrhythmias, primary peripheral vascular disease, cardiopulmonary dysfunction, and intraabdominal hypertension, as well as those who may be jeopardized by fluid challenge test, were excluded.

### Study protocol and ultrasonography measurements

On arrival at the AICU immediately after the operation, the patients were sedated and mechanically ventilated in a volume-controlled mode with the following parameters: tidal volume of 8 ml kg^−1^ at a respiratory rate of 12 breaths min^−1^, the inspiratory to the expiratory ratio of 1:2 and the positive end-expiratory pressure was set with 5 cmH_2_O before ultrasound assessment. Patients were sedated with continuous intravenous infusion of propofol at the rate of 1 mg kg^−1^ h^−1^, and dexmedetomidine 0.2 ug kg^−1^ h^−1^. During the study period, the sedative drugs, as well as ventilation settings, remained constant. All hemodynamic variables were collected, and ultrasound examination and measurements were performed before and after the fluid challenge, using 5 ml kg^−1^ of compound sodium chloride infused within 15 min. According to the changes in cardiac output (CO) increased ≥ 15% or not, patients were classified as responder group or non-responder group.

All transthoracic ultrasound measurements were performed by a proficient investigator in echocardiography using EPIQ 7C machine (Philips Healthcare, Andover, Massachusetts) equipped with an S5-1 probe. All Doppler echocardiographic measurements were taken offline from videotape recordings. Another investigator performing measurement was blinded to the patients’ hemodynamic parameters.

**Fig. 1:**
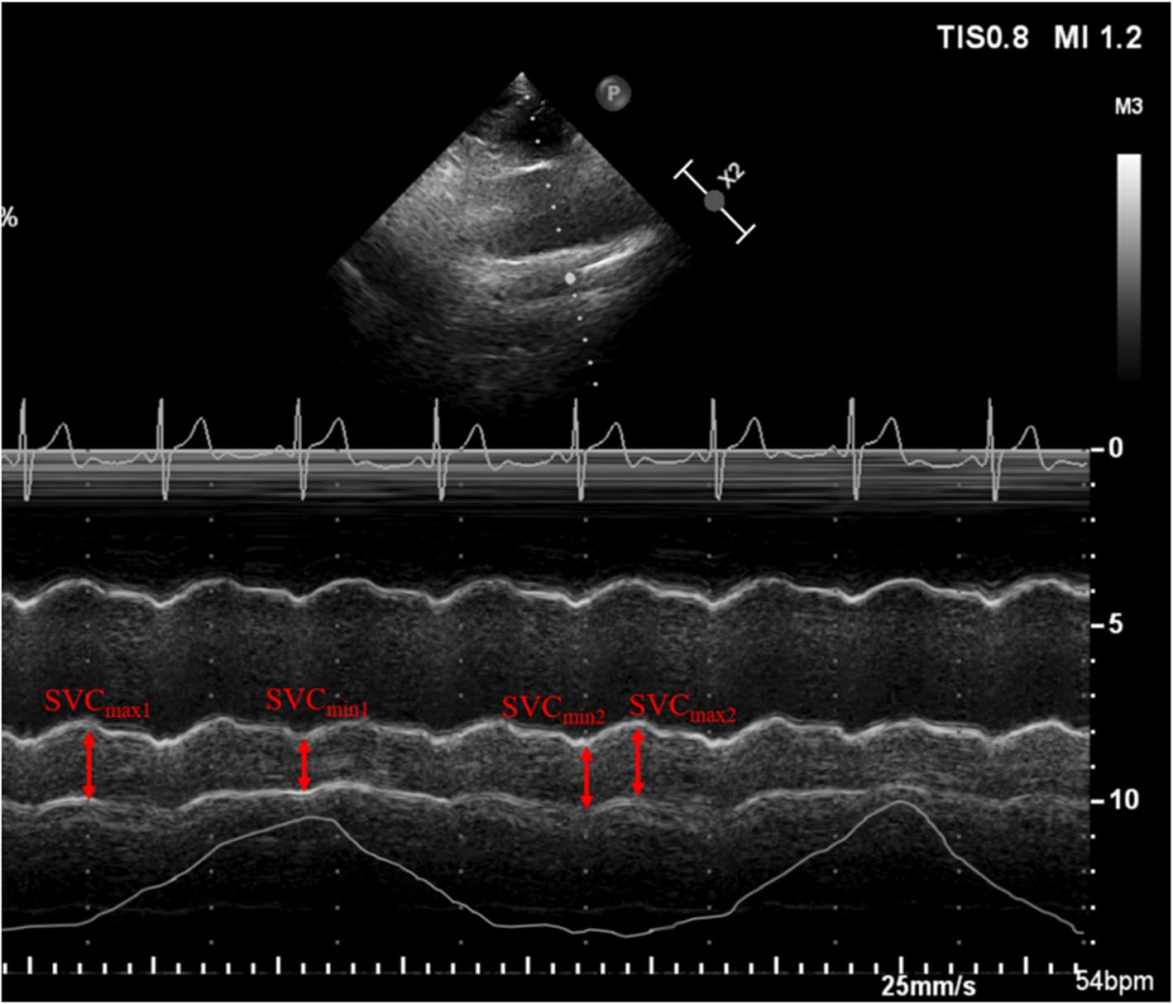
M-mode visualization of the superior vena cava (SVC) measured with transthoracic echocardiography showing the maximal (SVCmax_1_) and minimal (SVCmin_1_) diameters over the respirophasic cycle and maximal (SVCmax_2_) and minimal (SVCmin_2_) diameters over the cardiac cycle

The longitudinal axis of SVC was obtained from a left parasternal view using the two-dimensional view according to the methodology described and validated in previous studies by Ugalde D et al [12], with the patients in a semi-recumbent position. Pulse wave Doppler and visualization of a central venous catheter were used to differentiate the SVC from nearby structures. The diameter of SVC under different periods was assessed using an M-mode modality. The SVC diameter over a single respiratory cycle (SVCmax_1_, SVCmin_1_) was measured, and SVCCI was calculated as SVCCI = (SVCmax_1_-SVCmin_1_) / SVCmax_1_. According to the electrocardiogram anonymously recorded on the echocardiography images, the maximum (SVCmax_2_) and minimum (SVCmin_2_) diameter over the cardiac cycle were recorded, and SVCV was computed as follows: SVCV = (SVCmax_2_-SVCmin_2_) / SVCmax_2_ (Fig. 1). The whole SVC scan procedure took less than 10 min.

To achieve the longitudinal axis of inferior vena cava (IVC), the phased-array ultrasound probe was positioned in the right mid-axillary line at the level of the diaphragm with the probe marker towards cephalad. The maximum IVC diameter (IVCmax) on inspiration and the minimum IVC diameter (IVCmin) on expiration during a respiratory cycle were measured 2–4 cm distal the cavo-atrial junction under M-mode modality. The distensibility of IVC under mechanical ventilation was calculated using the equation: dIVC = (IVCmax-IVCmin)/IVCmin.

After achieving the velocity–time index (VTI) of left ventricular outflow tract (LVOT) blood flow with pulsed-wave Doppler in an apical five-chamber view and the diameter (D) of LVOT from the parasternal long-axis view at the level of aortic valve annulus in early systole, CO can be then evaluated using the formula CO = 3.14 × (D × 2^–1^)^2^ × VTI × HR. Each measurement was performed three times, and the average was used in the final analysis to reduce the statistical error.

### Statistical analysis

The results of our preliminary study of 25 patients showed that the AUC for SVCCI or dIVC to predict fluid responsiveness under mechanical ventilation was 0.938 and 0.80, respectively. The result showed that at least 66 patients were required to detect the difference of 0.138 between the AUC of SVCCI (0.938) and dIVC (0.8), with a power of 0.8 and a two-tailed type I error of 0.05, assuming the fluid responsiveness incidence of 50% in postoperative patients. To allow for a possible 20% dropout rate, a sample size of 79 was used.

The Shapiro–Wilk test was performed to test the normal distribution of variables. Numerical data were expressed as mean (SD) or median (interquartile range) where appropriate. Hemodynamic and echocardiographic parameters at baseline between two groups were compared using an independent t-test or the Mann–Whitney U test. The effects of fluid loading on variables within groups were studied with a paired t-test or Wilcoxon test. The Pearson and Spearman correlation coefficient was used to assess the relationships of the percentage change in CO with vena cava-related parameters. Receiver operating characteristic (ROC) curve analysis was performed to determine the efficiency of vena cava-related parameters in predicting fluid responsiveness, and the areas under the ROC curve (AUC) with a 95% confidence interval (CI) were calculated. The optimal cut-off value was determined by the point of the maximum Youden index. A comparison of AUCs between two indicators was conducted following the methodology described by Delong and his colleagues [13]. The gray zone approach was performed to determine an uncertain range of predictive parameters. Software SPSS (version 23.0, USA) and Medcalc 19.6.1 (version 19.6.1, Belgium) were employed for statistical analysis. A *P*-value less than 0.05 was considered statistically significant.

## Results

Seventy-nine patients were assessed for eligibility during the study period. A total of seventy patients were included in the final analysis, whereas nine patients were excluded because of poor SVC image quality. Of those, there were 30 responders (42.9%) according to the criterion of the fluid challenge test.

Baseline demographic characteristics of the patients are presented in Table 1. There was no statistical difference in the demographic variables or intraoperative data between patients who responded to volume expansion and those who did not (*P* > 0.05).

**Table 1:** Baseline demographic and intraoperative data of patients responding to fluid challenge or not

Variable	All patients (*n* = 70)	Responder (*n* = 30)	Non-responder (*n* = 40)	*P* value
Age (yr)	61 (52–68)	61 ± 9	63 (51–70)	0.891
Female (n, %)	34 (48.6)	18 (60)	16 (40)	0.098
BMI (kg m^−2^)	23.24 ± 1.64	23.14 ± 1.61	23.32 ± 1.69	0.668
BSA (m^2^)	1.65 ± 0.13	1.62 ± 0.12	1.67 ± 0.13	0.164
ASA (III, %)	59 ± 84.3	27 ± 90	32 ± 10	0.420
Medical history				
Hypertension	19 (27.1)	14 (46.7)	15 (37.5)	0.441
Diabetes mellitus (n, %)	10 (14.3)	6 (20)	4 (10)	0.402
History of coronary artery disease (n, %)	2 (2.9)	0 (0)	2 (5)	0.503
History of cerebrovascular artery disease (n, %)	5 (7.1)	3 (10)	2 (5)	0.645
Surgey duration (h)	3.46 (2.71–4.23)	3.63 (2.75–4.81)	3.42 (2.62–4.06)	0.458
Fluid infusion (ml)	2000 (2000–2600)	2250 (2000–2850)	2000 (1575–2500)	0.224
Estimated blood loss (ml)	200 (100–300)	200 (100–325)	200 (100–300)	0.775
Urine output (ml)	500 (275–800)	600 (200–938)	400 (300–650)	0.220
Use of intraoperative vascular active agents (n, %)	13 (18.6)	4 (13.3)	9 (22.5)	0.329
Types of surgery				0.093
Radical resection of hepatobiliary and pancreatic tumors	29 (41.4)	15 (50)	14 (35)	
Radical resection of gastrointestinal tumors	25 (35.7)	11 (36.7)	14 (35)	
Radical resection of urological tumors	11 (15.7)	2 (6.7)	9 (22.5)	
Others	5 (7.1)	2 (6.7)	3 (7.5)	

*BMI* Body mass index, *BSA* Body surface area, *ASA* American Society of Anesthesiologists physical status

As shown in Table 2, the responders had a larger SVCCI, dIVC, and SVCV (*P* < 0.05) compared to non-responders at baseline. Baseline SVCCI significantly correlated with the change in CO (ΔCO) (*r* = 0.606, *P* < 0.0001), while dIVC and SVCV had weak correlation with ΔCO (*r* = 0.354, *P* = 0.003; *r* = 0.321, *P* = 0.007).

**Table 2:** Main hemodynamic and ultrasonographic parameters between responders and non-responders at baseline and after volume expansion

Parameters	Time Point	Resonder (*n* = 30)	Nonresponder (*n* = 40)	*P*
MAP (mmHg)	T0	91 ± 13	90 ± 11	0.829
	T1	94 ± 15	92 ± 11	0.506
CVP (cmH_2_O)	T0	6 ± 2	6 ± 2	0.848
	T1	7 ± 2†	7 ± 3†	0.774
HR (beats min^−1^)	T0	62 ± 11	60 ± 9	0.346
	T1	64 ± 10†	59 ± 8†	0.013
VTI (cm)	T0	20.8 ± 3.4	22.3 ± 3.3	0.054
	T1	24.3 ± 3.4†	23.3 ± 3.7†	0.286
CO (L min^−1^)	T0	3.9 (3.5–4.6)	4.3 ± 1.0	0.455
	T1	4.7 (4.2–5.6) †	4.4 ± 1.0†	0.02
SVCCI	T0	0.33 ± 0.11	0.16 (0.12–0.21)	< 0.001
	T1	0.29 ± 0.09	0.17 ± 0.08	< 0.001
dIVC	T0	0.20 (0.16–0.35)	0.13 (0.08–0.21)	0.001
	T1	0.22 (0.14–0.33)	0.12 (0.07–0.19)	0.002
SVCV	T0	0.18 (0.13–0.29)	0.11 (0.07–0.15)	< 0.001
	T1	0.20 (0.14–0.28)	0.13 ± 0.08	< 0.001

^†^*P* < 0.05 versus before fluid challenge test. T0, Before fluid challenge; T1, After fluid challenge; *P*-value corresponds to the comparison between Responders and Nonresponders

After the fluid challenge, significant increases in CVP, VTI, and CO in both groups were observed (*P* < 0.05). In addition, there was an increase in heart rate (HR) in responders but a decrease in HR in nonresponders (*P* < 0.05). No statistical differences in the SVCCI, dIVC and SVCV were noted before and after fluid loading, respectively (*P* > 0.05).

**Fig. 2:**
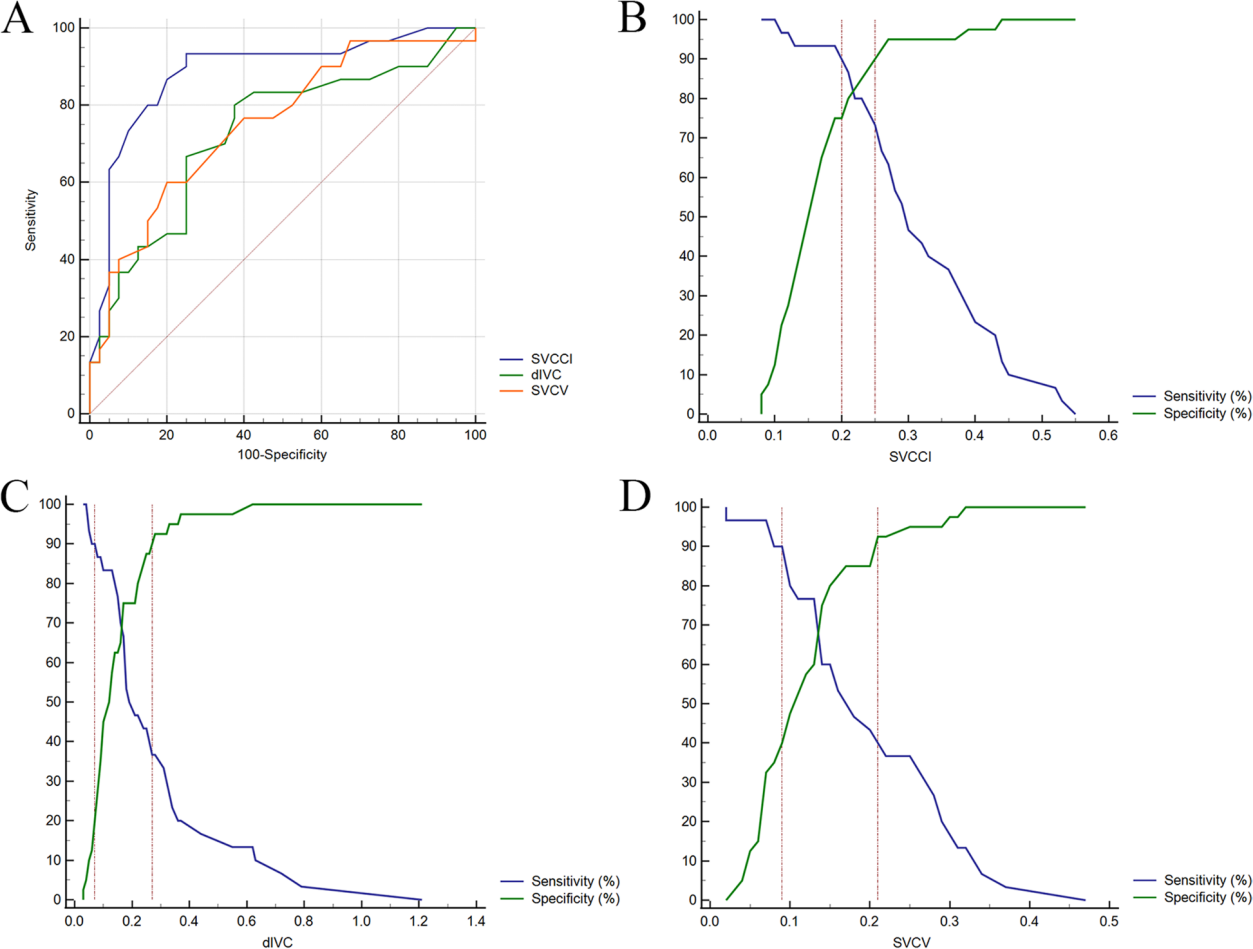
Receiver operating characteristic curves comparing the ability of superior vena caval collapsibility index (SVCCI), inferior vena cava distensibility index (dIVC), and superior vena caval variation over the cardiac cycle (SVCV) (**A**) to discriminate responders from nonresponders. Sensitivity and specificity plots predict fluid responsiveness according to the values of SVCCI (**B**), dIVC (**C**), and SVCV (**D**) to determine the gray zone which is indicated by the two vertical dotted lines

SVCCI by thoracic ultrasonography was a great predictor of fluid responsiveness, with an AUC of 0.885 (95% CI, 0.786–0.949; *P* < 0.0001). The optimal cut-off value for SVCCI was 19%, with a sensitivity of 93.3% and a specificity of 75% (Table 3). To avoid the dualism of a single cutoff value, a sensitivity/specificity plot shows the probability interval for SVCCI. The gray zone for SVCCI lay between 20 and 25% and comprising only 11 (15.7%) of patients (Fig. 2).

**Table 3:** Diagnostic capability of dynamic parameters to predict fluid responsiveness

Parameters	AUC curve (95% CI)	*P*-value	Optimal cut-off value	Sensitivity (%) (95% CI)	Specificity (%) (95% CI)	Youden index	Gray zones	Patients in gray zones (%)
SVCCI	0.885 (0.786–0.949)	< 0.0001	> 0.19	93.3 (77.9–99.2)	75 (58.8–87. 3)	0.683	0.2–0.25	11 (15.7)
dIVC*	0.727 (0.608–0.827)	0.0003	> 0.14	80 (61.4–92.3)	62.5 (45.8–77.3)	0.425	0.07–0.27	44 (62.9)
SVCV*	0.751 (0.633–0.847)	< 0.0001	> 0.15	60 (40.6–77.3)	80 (64.4–90.9)	0.4	0.09–0.21	35 (50)

^*^SVCCI versus dIVC, *P* = 0.0369; SVCCI versus SVCV, *P* = 0.0372

An SVCV > 15% obtained from the study allowed us to discriminate between the responders and the non-responders with a sensitivity of 60% and specificity of 80%, respectively. However, dIVC and SVCV were inferior predictors of fluid responsiveness with an AUC of 0.727 (95% CI, 0.608–0.827; *P* < 0.001) and 0.751 (95% CI, 0.633–0.847; *P* < 0.0001), which were significantly lower than the AUC for SVCCI (*P* < 0.05), respectively. Besides AUC, the gray zone of dIVC (7%-27%) and SVCV (9%-21%) includes 62.9% and 50% of patients, respectively (Fig. 2).

## Discussion

To our knowledge, this is the first study to compare the value of diameter variation of SVC during respiratory or cardiac phase with dIVC using noninvasive TTE for predicting fluid responsiveness in postoperative mechanically ventilated patients. We found that SVCCI had a more significant correlation coefficient with ΔCO, a larger AUC, and a smaller gray zone than dIVC and SVCV, indicating that the respiratory variation of SVC is superior to dIVC and SVC cardiac variation in predicting volume responsiveness.

The cyclic effect of positive pressure ventilation on the changes in vena cava diameter depends on the transmural pressure, which is determined by intravascular pressure (circulating volume and right heart function) and surrounding pressure (intrathoracic pressure for SVC and abdominal pressure for IVC) [14]. IVC respiratory variation has consistently been recommended to assess potential intolerance to fluid administration [15]. However, conflicting results have been published concerning about the reliability of this index [16, 17]. According to the previous research, our trial results showed that a moderate diagnostic accuracy when using dIVC as the AUC for predicting fluid responsiveness was 0.727 (95% confidence interval, 0.608 to 0.827; *P* < 0.001). A dIVC > 14% discriminates responders from non-responders with a sensitivity of 62.5% and specificity of 80%, respectively. However, the gray zone of dIVC (7%-27%) includes more than half of the patients, suggesting that dIVC was not clinically useful in mechanically ventilated patients after abdominal surgery which may be inflicted by multiple factors including the intra-abdominal pressure, intrathoracic pressure, and pericardial pressure as well.

Compared with IVC, SVC is located entirely in the thoracic cavity, so it is hardly affected by intra-abdominal pressure [11, 18]. Vieillard Baron et al. have initially found that respiration-induced SVC cyclic collapse reflects insufficient venous filling and can be corrected by volume expansion [19]. In addition, SVCCI can accurately predict the fluid responsiveness in those false-negative patients diagnosed with PPV due to the presence of severe acute cor pulmonale [20]. In a large cohort, Vignon P et al. compared several dynamic echocardiographic parameters used to predict fluid responsiveness and reported that the AUC for SVCCI (0.75) was significantly greater than those both for dIVC (0.63) and PPV (0.67) in patients with mechanical ventilation [10]. The best cut-off value for SVCCI was 21% (61% sensitivity, 84% specificity) which concurred with ours.

In the existing studies, however, the SVC measurement was available in almost every ventilated patient by TEE [21]. Ugalde D and his colleagues recently provided a practical method of imaging SVC from a left parasternal view in its longitudinal axis and proved its feasibility in critically ill patients [12]. In our study, we excluded 9 (11.4%) subjects whose SVC views were not obtainable, which is in line with the pilot study conducted by Ugalde D et al. We concluded that an SVCCI ≥ 19% measured with TTE predicted responders correctly with sensitivity and specificity of 93.3% and 75% respectively. The AUC for SVCCI was 0.885, which was as high as previously reported. As regard to a relatively low cut-off value, the discrepancies may be explained by the different study populations, measurement methods, fluid loading strategies, and fluid responsiveness standards [18]. Furthermore, we performed the gray zone approach, which was suggested by Cannesson et al [22] to test the clinical utility to avoid dichotomizing the results. For SVCCI, there were only 15.7% of patients in the gray zone which underlined the robustness of SVCCI. The better predictive value of SVCCI could be attributed to a relatively greater decrease in venous return in the thoracic cavity caused by the mechanical insufflation than the peritoneal cavity.

Cardiac variation in the great veins was verified to be caused by venous return, driven by the systemic filling pressure rather than by pressure propagation from the aorta [23–25]. Therefore, it is useful to assess the venous volume with a cardiac variation. The IVC cardiac variation was first described by Nakamura, who applied engineered programs to trace IVC automatically and found that IVC cardiac variation is related to volume status [23]. SVC cardiac variation is more apparent, and easily measurable compared with IVC [25]. The study by Zhi Cheng et al. [11] proved that SVC cardiac variation could predict preload responsiveness with the AUC of 0.849 in patients receiving mechanical ventilation. The optimal cut-off value was 21.1%, with sensitivity and specificity of 76.9% and 84.8%. Consistent with the previous results, SVCV can estimate fluid responsiveness accurately but with a relatively larger gray zone, laying between 9 and 21%, and contained 35 (50%) patients in the present study. Nevertheless, our results showed that the diagnostic value of SVCCI is higher than that of SVCV regardless of in the aspects of AUCs or the proportion of patients in the gray zone. Similarly, Sonoo T et al [26] discovered that the correlation between cardiac variation and respiratory variation in IVC was low, indicating that the effectiveness of cardiac variation as an indicator of fluid responsiveness is limited.

Although our present study has highlights, several limitations have to be addressed. First, we did not monitor the intra-abdominal pressure directly. The population we investigated is patients after abdominal surgery whose abdominal pressure may be abnormal. The monitoring of bladder pressure in all patients is not a routine in our institution. Secondly, although the golden standard to evaluate the effect of CO is pulmonary artery catheterization, echocardiography has been validated to correlate well with this invasive method [27]. Thirdly, all measurements were achieved in mechanically ventilated patients, and our conclusion may not be extrapolated to spontaneously breathing patients. Fourth, we did not acquire the SVC image by the conventional TEE approach to appraise the correlation between the SVC-derived parameters. However, the transthoracic acquisition of SVC was equivalent to the TEE approach in image quality and measurement [12].

## Conclusions

Superior vena cava-related parameters measured by noninvasive transthoracic echocardiography have higher diagnostic effectiveness in predicting fluid responsiveness in mechanically ventilated patients after major abdominal surgery. Especially, the SVCCI is superior to the conventional variables of dIVC and SVCV in terms of the prediction reliability and the proportion of subjects in the gray zone. Therefore, the SVCCI assessment by the surface thoracic ultrasound should be advocated in mechanically ventilated patients undergoing major abdominal surgery.

## Data Availability

All data generated or analyzed during this study are included in this published article.

## Abbreviations

AICU: Anesthesia intensive care unit
ASA: American Society of Anesthesiologists physical status
AUC: Area under the receiver operating characteristic
BMI: Body mass index
CI: Confidence interval
CO: Cardiac output
CVP: Central venous pressure
dIVC: Inferior vena cava distensibility index
HR: Heart rate
IVC: Inferior vena cava
IVCmax: Maximum IVC diameter
IVCmin: Minimum IVC diameter
LVOT: Left ventricular outflow tract
MAP: Mean arterial pressure
PPV: Pulse pressure variation
ROC: Receiver operating characteristic
SVC: Superior vena cava
SVCCI: Collapsibility index of SVC
SVCV: SVC variation over the cardiac cycle
SVCmax_1_: Maximum SVC diameter over respiratory cycle
SVCmin_1_: Minimum SVC diameter over respiratory cycle
SVCmax_2_: Maximum SVC diameter over the cardiac cycle
SVCmin_2_: Minimum SVC diameter over the cardiac cycle
SVV: Stroke volume variation
TEE: Transesophageal echocardiography
TTE: Transthoracic echocardiography
VTI: Velocity time integral
